# Current Status of Standardization of Traditional Chinese Medicine in China

**DOI:** 10.1155/2016/9123103

**Published:** 2016-03-27

**Authors:** Juan Wang, Yi Guo, Gui Lan Li

**Affiliations:** ^1^Baokang Hospital, Tianjin University of Traditional Chinese Medicine, No. 88, Yuquan Road, Nankai District, Tianjin 300193, China; ^2^Plot Construction Unit of Acupuncture and Moxibustion and Standardization Research Center of State Administration of Traditional Chinese Medicine, Tianjin 300193, China; ^3^Acupuncture and Moxibustion College, Tianjin University of Traditional Chinese Medicine, No. 312, Anshan West Road, Nankai District, Tianjin 300193, China

## Abstract

As an important component of traditional medicine, Traditional Chinese Medicine (TCM) is widely spread and applied in more than 100 countries across the world. The standardization of TCM is very important for the international application of Chinese medicine. In this paper, we have explained and analyzed the standardization situations of TCM in China with the purpose of providing reference for standardization and international development of TCM.

## 1. Introduction

TCM has been widely applied to prevention and treatment for various diseases from ancient times till now [1]. TCM, a natural therapy, is safe, effective, and widely used and is increasingly attracting worldwide attention [2].* WHO Traditional Medicine Strategy: 2014–2023* released by the World Health Organization shows that TCM has spread to more than 100 countries and has grown into an international industry [3]. However, due to the lack of relevant standards, the development of TCM is undermined and the progress of internationalization and modernization of TCM is seriously influenced [4, 5].

As the country of origin and application of TCM, China has a unique TCM theoretic system and effective treatment methods. In recent years, after the constant transformation of concepts and methods, great breakthroughs and remarkable achievements have been made in terms of standardization of TCM, but some problems remain [6]. In this paper, we mainly focus on summary and analysis on research of current situations in standardization of TCM (excluding acupuncture and moxibustion and traditional Chinese herbs) [7].

## 2. Current Status of Standardization of Traditional Chinese Medicine in China

### 2.1. China Attaches Great Importance to Standardization of TCM

Standardization of TCM, under the principle of “unification, simplification, coordination, and optimization” and by its comprehensive use, refers to developing and applying standards to the various links, processes, and objects of TCM such as medical care, scientific research, education, industry, culture, and management, to promote academic development and propagation of achievement of TCM, to standardize management of TCM, to guarantee the quality and safety of TCM, to promote the modernization and international communication of TCM so as to achieve good economic and social benefits, and to achieve guiding and supporting a series of activities for the purpose of the comprehensive development of TCM [8]. Standardization of TCM is an essential part of the modernization of TCM. In 2009, the State Council of China issued* Several Opinions of the State Council on Supporting and Promoting the Development of Traditional Chinese Medicine*, in which “promoting the standardization of TCM” was included.

In the National Work Conference on Traditional Chinese Medicine convened in 2011, Comrade Li Keqiang, a member of Central Politburo Standing Committee of the Communist Party of China and Vice Premier of the State Council, stressed that “the construction for the standardization of TCM should be strengthened.”

In the* Twelfth Five-Year Planning Outline for National Economic and Social Development of the People's Republic of China*, National Development and Reform Commission has included “promotion of standardization and normalization of TCM” as one of the important tasks. Ministry of Science and Technology took the lead to formulate* Traditional Chinese Medicine Innovation Development Planning Outline 2006–2020*, in which the standardization of TCM was set as one priority area for being the standard specifications of the international traditional medicine. Standardization Administration of the People's Republic of China will support the TCM as an important field for international breakthrough and arrange almost hundreds of standard items of TCM in the service standard development plan.

### 2.2. Standard System of TCM Has Been Basically Established in China

During the “Eleventh Five-Year Plan” period, the TCM standard system has been basically established. The system consists of four categories of standards, namely, basic standards, technical standards, management standards, and working standards [9], and formulation and revision of basic standards and technical standards were stressed.

### 2.3. Current Status of the Formulation and Revision of TCM Basic Standards in China

The formulation of basic and general standards of Traditional Chinese Medicine started with the focus on formulation and revision for basic theoretical terminology, terminology of some subjects of TCM and their classification and codes, and other standards to achieve TCM terminology standardization. Five national standards of TCM have been issued in succession, namely,* Classification and Codes of Diseases and ZHENG of Traditional Chinese Medicine* (GB/T15657-1995) as China's first national standard for classification of TCM diseases stipulates the classification principles and coding methods of diseases and ZHENG of TCM and has the corresponding computer software, promoting the standardization of TCM clinical diagnosis;* Clinic Terminology of Traditional Chinese Medical Diagnosis and Treatment-Diseases* (GB/T 16751.1-1997),* Clinic Terminology of Traditional Chinese Medical Diagnosis and Treatment-Syndromes* (GB/T 16751.2-1997), and* Clinic Terminology of Traditional Chinese Medical Diagnosis and Treatment-Therapeutic Methods* (GB/T 16751.3-1997) specify the basic terminology of common diseases, syndromes, and therapeutic principles of TCM to improve the medical quality of TCM and the level of scientific management of medical institutions of TCM positive role and* Basic Theory Terminology of Traditional Chinese Medicine* (GB/T 20348-2006) provides the basic standard for the terminology of TCM theories such as Yin and Yang, five elements, and main and collateral channels, which is of great significance for the inheritance and innovation of TCM and meeting the needs of the teaching, scientific research, health care, management, and foreign exchange of TCM [10].

### 2.4. Current Status of the Formulation and Revision of TCM Technical Standards in China

The clinical practice of TCM needs not only the guidance of TCM theory but the tools of medical treatment. From the 1980s to 2002, TCM authorities have issued nearly 70 technical standards such as* Criteria of Diagnosis and Therapeutic Effect of Diseases and Syndromes in TCM* (ZY/T001.1-94) and* Clinical Guidelines for Emergency Treatment of Internal Medicine of Traditional Chinese Medicine* (YY228), and TCM academic societies have issued over 50 technical specifications [6].* Criteria of Diagnosis and Therapeutic Effect of Diseases and Syndromes in TCM* is the first industry standard of TCM in China for 9 clinical departments with 406 diseases and syndromes, including internal medicine, surgery, gynecology, and pediatrics, marking that the major progress was made in the standardization of TCM industry in China.

After 2002, the formulation of industry technical standards was further strengthened, prevention and treatment of common diseases and major diseases were mainly focused on and diagnostic and treatment guidelines for clinical syndromes in TCM, and operation regulation of TCM clinic technology and others were formulated and revised as the priority. Completed standards by the State Administration of Traditional Chinese Medicine include 18 standards for the series of* Routine and Technical Operation Regulation of Nursing Care in TCM* (ZYYXH/T 1.1-2006~ZYYXH/T 1.18-2006) for nursing care of clinical departments such as internal medicine, surgery, gynecology, and pediatrics, 19 standards for the series of* Technical Specification of Health Preservation and Prevention of Chinese Medicine* (ZYYXH/T 158-2010~ZYYXH/T 176-2010) with massage on the spine, the whole body, pediatrics and moxibustion, medicinal liquor, acupoint sticking, cupping, and so on to guide and standardize technical operations of TCM health care of care physicians and technicians and make technical operations more standardized and more safe, 1 standard of* Classification and Determination of Constitution in TCM* (ZYYXH/T 157-2009) as the important reference basis for the clinical practice, to determine the specification and quality evaluation, 10 standards for the series of* Standard Specifications for Study and Compilation of Traditional Chinese Medicine Classics* (ZYYXH/T 362-2012~ZYYXH/T 371-2012) for proofreading TCM classics, 1 standard of* Clinical Guidelines of Chinese Medicine on Sub-Health* (ZYYXH/T 2-2006) for clarifying the understanding of subhealth of TCM, common syndromes, and intervention principles of TCM on subhealth to gain the scientific, standardized diagnosis and intervention on subhealth and provide evidences for the feasible health management plan and intervention measures on subhealth [11], 15 standards for the series of* Guidelines for TCM Diabetes Prevention and Treatment* (ZYYXH/T 3.1-2007~ZYYXH/T 3.15-2007) for the course, complications, and merged diseases of diabetes to have detailed and normative prevention and treatment strategy of TCM, achieving the standardized, scientific prevention and treatment of TCM of diabetes [12], 21 standards for the series of* Guideline for Diagnosis and Treatment of Tumor in TCM* (ZYYXH/T 136-2008~ZYYXH/T 156-2008) for the standard of differentiation of syndromes and signs for classification and diagnosis and treatment of TCM of common malignant tumor (according to preliminary clinical statistics, 70–80% of cancer patients accepted the different levels of TCM treatments; Chinese medicine tumor has become an important part of modern Chinese medicine clinical disciplines; this “guideline” can improve the overall level of TCM prevention and treatment of malignant tumor [13]), and 386 standards for the series of* Guideline for Diagnosis and Treatment of Common Diseases in TCM*. In total, 397 diseases of 11 clinical majors were involved such as internal medicine, surgery, gynecology, pediatrics, ophthalmology, ENT, anorectal section, dermatology, and orthopedics, and the most common TCM clinical diseases were covered [9]. Three systems, namely, technical specification for clinical diagnosis and treatment of common TCM diseases, health care technology, and nursing care, have been formulated and the criteria for clinical diagnosis and treatment in TCM are further enriched.

### 2.5. Current Status of the Formulation and Revision of TCM Management Standards in China

TCM management standards are formulated to regulate the TCM industry and control matters required for uniform coordination in the management practice.

The State Administration of Traditional Chinese Medicine has combined with relevant departments to issue many management standards related to construction of traditional Chinese hospitals, construction of major TCM laboratories, classification of TCM research laboratories, TCM education management, and others [14] and covered TCM treatment, scientific research, education, and other fields, thereby laying foundation for the construction of TCM standard system.

In summary, 5 national TCM standards and over 480 TCM industrial standards have been formulated and issued for implementation and the TCM standard system is primarily established, which ensures the standardization of TCM development smoothly, and improves the standard for formulation and revision work. See the publication situations of current TCM standards in Table 1.

### 2.6. TCM Standardization Support System Has Been Basically Established

The support system is to promote and guarantee TCM standards, including management system, operation system, technical platform, professional team, implementation and guarantee, inspection and evaluation, and information service.

### 2.7. TCM Standardization Management System and Operation Mechanism Have Been Primarily Established

TCM Standardization Management Department of China Administration of Traditional Medicine is responsible for centralized management and coordinated planning of TCM standardization. The standardization office is specially built as the routine administrative office for TCM standardization to fully coordinate the standardization of the industry.

Several TCM standardization technology research centers have been established across the country to strengthen the organizational management on formulation, implementation, and supervision of TCM standards [15]. In September 2010, China Administration of Traditional Medicine confirmed 42 base (pilot) construction units for TCM standardization research and promotion so as to provide a strong guarantee for implementation and promotion of TCM standards. China Administration of Traditional Medicine has released* TCM Standard Formulation Procedure* and* Interim Measures of State Administration of Traditional Medicine on Management of TCM Standardization Project*, in which the formulation and management procedure of TCM standards have been regulated [16].

### 2.8. TCM Standardization Technology Team Has Been Basically Formed

Talents are essential to promote the TCM standardization. With the constant establishment of TCM standardization research institutes and academic communities, the TCM standardization talent team is gradually expanding from scratch.

In 2006, the first National TCM Standardization Training Class was held in Wuhan and attracted over 120 TCM professional participants across the country in the technical training; in 2008, China Administration of Traditional Medicine established the pilot construction unit of “Acupuncture and Moxibustion Research Center of China Administration of Traditional Medicine” in Tianjin University of Traditional Chinese Medicine and “TCM Technical Training and Research Center of China Administration of Traditional Medicine in Hubei University of Traditional Chinese Medicine,” thereby providing important intelligent support and sufficient guarantee for implementation of TCM standardization development plan; and by 2009, over 800 researchers and experts participated in standardization projects, initially forming the TCM standardization team of a certain size [17].

### 2.9. Actively Participate in TCM International Standardization

In 2003, the World Federation of Chinese Medicine Societies was established and headquartered in Beijing. It enhanced the formulation, publicity, and promotion of TCM international standards. In 2007, WHO issued* International Standard Terminologies on Traditional Medicine*, which was prepared in corroboration by personnel from China, Korea, Japan, and other countries.

In September 2009, International Organization for Standardization (ISO) established Committee of TCM Standardization Technology with the code of ISO/TC249 (tentatively) [18]. In ISO/TC249, a total of 44 standard projects have been put forward since 2011, including 29 projects by China [19].

Upon the revision of* Codes of International Classification of Diseases* (ICD-11), WHO has decided to include the traditional medicine, and China has actively participated in the preparation of traditional medicine. All of the works lay an important milestone for the international standardization of TCM and also are the important opportunities for China to regain the initiative in the international standardization.

## 3. Problems and Some Countermeasures of the Construction of TCM Standardization in China

Since 1980s, the constant exploration on the standardization of Traditional Chinese Medicine (TCM) has made certain achievements. However, on account of its long-term, arduous, and complex nature, the current TCM standardization still falls behind with the developmental needs of TCM.

### 3.1. Awareness of TCM Standardization Needs to Be Reinforced

Since TCM standardization is still in its developing phase and inadequate in publicizing and implementing, a number of TCM practitioners still hold weak consciousness and different cognitions towards TCM standardization, thus resulting in their infirm identity, urgency, and responsibility for carrying out TCM standardization [20]. Therefore, we should enhance publicity and education of TCM standardization to improve awareness of TCM practitioners and attach great importance to the synchronization from the clinics to the classrooms [21, 22] so as to pave the way for the future implementation of TCM standardization.

### 3.2. TCM Standardization System Needs to Be Improved

Published standardization at present revealed limited coverage in criteria of TCM, emphasizing the foundation and operation without covering in all areas. The application of the latest industry standards of seven clinical trials of TCM into clinical trials for 11 kinds of gynecology diseases has gained international recognition [23], which means it is very necessary to further revise the standards and to strengthen the standards on health care, research, education, information, especially management, and other areas of TCM. That will ensure a balanced development of the quantity, quality, structure, and benefit which make further efforts to enrich and improve the whole TCM standardization system [24].

### 3.3. Revised Methods of TCM Standardization Needs to Be Completed

Many of the present standardizations are mainly based on the literatures and lack of evidence-based medicine and clinical evidence, which impede their execution in clinical practice [25]. Moreover, the defective revising procedure gives rise to the “aging” of TCM standardization [20]. For instance, ever since the publication of the national standard,* Clinic Terms of Traditional Chinese Medicine* in 1997, it has not yet been revised till now. As a consequence, the lagging update of standards makes them detached with reality and thus unable to truly reflect the current academic level and management requirements of TCM, which hinders the popularization and application of TCM standards [26].

For the present situation, methodology researches of the standard revision need to be improved to reach a redaction method suitable for its own features of TCM, which will perform a positive effect on TCM standardization.

### 3.4. Consolidate the Support System of TCM Standardization

Development of TCM standards is witnessing booming growth, while the progress of related support system is comparatively slow, which is still in its infancy and in need of further refining. So far, the management, implementation, and supervision system of TCM standardization is still incomplete; surveys and feedbacks of its applicability are also imperfect; what is more, it also shows a shortage of talented professionals in such a field [16]. In 2012, the State Administration of Traditional Chinese Medicine issued* the Guidance on Strengthening the Standardization of Traditional Chinese Medicine*, under whose direction support system of TCM standardization is bound to receive a much more comprehensive and orderly development.

### 3.5. Vigorously Advance the Process of TCM International Standardization

Most of the present established TCM standards are domestic, with few international ones. So it is important to take full advantage of China's status as a permanent member of international standardization organization as well as its working role in secretariat of TCM technical committee, so as to play an active part in setting development strategies, regulations, and projects of TCM standardization; besides, dominance of TCM international standardization should be seized in order to promote the transformation of TCM standards to international ones [27].

The standardization of TCM is a gradual process, as well as a course of innovation and academic progress. Because of its fundamental, strategic, and global effects in development of TCM, the standardization should be promoted under a comprehensive plan and top design and proceeded with phased and planned steps [28].* The Medium- and Long-Term Development Plan about the Standardization of Traditional Chinese Medicine (2011–2020)* has been published, and we believe, under its direction, there will be a more scientific and orderly development of TCM standardization for the services of people's health.

## Figures and Tables

**Table 1:** The existing standards of Traditional Chinese Medicine in China (a total of 486).

	Serial number	Standard number	Standard title	Issuance date	Execution date	Release unit
National standard (a total of 5)	1	GB/T15657-1995	*Classification and Codes of Diseases and ZHENG of Traditional Chinese Medicine*	25 July 1995	1 January 1996	AQSIQ; SAC
	2	GB/T 16751.1-1997	*Clinic Terminology of Traditional Chinese Medical Diagnosis and Treatment-Diseases*	4 March 1997	1 October 1997	AQSIQ; SAC
	3	GB/T 16751.2-1997	*Clinic Terminology of Traditional Chinese Medical Diagnosis and Treatment-Syndromes*	4 March 1997	1 October 1997	Chinese technical supervision bureau
	4	GB/T 16751.3-1997	*Clinic Terminology of Traditional Chinese Medical Diagnosis and Treatment-Therapeutic Methods*	4 March 1997	1 October 1997	Chinese technical supervision bureau
	5	GB/T 20348-2006	*Basic Theory Terminology of Traditional Chinese Medicine*	25 May 2006	1 October 2006	Chinese technical supervision bureau

Industry standard (a total of 481)	6	YY228	*Clinical Guidelines for Emergency Treatment of Internal Medicine of Traditional Chinese Medicine *	1 March 1994	1 March 1994	SATCM
	7	ZY/T001.1-94	*Criteria of Diagnosis and Therapeutic Effect of Diseases and Syndromes in TCM*	11 October 1994	1 January 1995	SATCM
	8	ZYYXH/T 2-2006	*Clinical Guidelines of Chinese Medicine on Sub-Health*	10 December 2006	10 December 2006	CACM
	9	ZYYXH/T1.1~1.18-2006	*Routine and Technical Operation Regulation of Nursing Care in TCM *	25 December 2006	25 December 2006	CACM
	10	ZYYXH/T3.1~3.15-2007	*Guideline for TCM Diabetes Prevention and Treatment*	28 July 2007	28 July 2007	CACM
	11	ZYYXH/T4~49-2008	*Guidelines for Diagnosis and Treatment of Common Internal Diseases in Chinese Medicine Symptoms in Chinese Medicine*	22 July 2008	22 July 2008	CACM
	12	ZYYXH/T50~135-2008	*Guidelines for Diagnosis and Treatment of Common Internal Diseases in Chinese Medicine Diseases of Modern Medicine*	22 July 2008	22 July 2008	CACM
	13	ZYYXH/T136~156-2008	*Guidelines for Diagnosis and Treatment of Tumor in Traditional Chinese Medicine*	30 November 2008	1 December 2008	CACM
	14	ZYYXH/T157-2009	*Classification and Determination of Constitution in TCM*	26 March 2009	9 April 2009	CACM
	15	ZYYXHT158~176-2010	*Technical Specification of Health Preservation and Prevention of Chinese Medicine *	23 December 2010	11 January 2011	CACM
	16	ZYYXH/T177~202-2012	*Guidelines for Diagnosis and Treatment of Common Diseases of Surgery in Traditional Chinese Medicine*	1 July 2012	1 August 2012	CACM
	17	ZYYXH/T203~246-2012	*Guidelines for Diagnosis and Treatment of Common Diseases of Gynecology in Traditional Chinese Medicine* ****	1 July 2012	1 August 2012	CACM
	18	ZYYXH/T247~286-2012	*Guidelines for Diagnosis and Treatment of Common Diseases of Pediatric in Traditional Chinese Medicine*	1 July 2012	1 August 2012	CACM
	19	ZYYXH/T287~306-2012	*Guidelines for Diagnosis and Treatment of Common Diseases of Otolaryngology in Traditional Chinese Medicine*	1 July 2012	1 August 2012	CACM
	20	ZYYXH/T307~321-2012	*Guidelines for Diagnosis and Treatment of Common Diseases of Otolaryngology in Traditional Chinese Medicine*	1 July 2012	1 August 2012	CACM
	21	ZYYXH/T322~341-2012	*Guidelines for Diagnosis and Treatment of Common Diseases of Coloproctology in Traditional Chinese Medicine*	1 July 2012	1 August 2012	CACM
	22	ZYYXH/T342~361-2012	*Guidelines for Diagnosis and Treatment of Common Diseases of Dermatology in Traditional Chinese Medicine*	1 July 2012	1 August 2012	CACM
	23	ZYYXH/T362~371-2012	*Standard Specification for Study and Compilation of Traditional Chinese Medicine Classics * ****	1 July 2012	1 August 2012	CACM
	24	ZYYXH/T372~415-2012	*Guidelines for Diagnosis and Treatment of Common Diseases of Orthopedics and Traumatology in Traditional Chinese Medicine*	1 July 2012	1 August 2012	CACM
	25	ZYYXH/T417~441-2012	*Guidelines for Diagnosis and Treatment of Common Diseases of Spinal Orthopedics in Traditional Chinese Medicine*	1 July 2012	1 August 2012	CACM

AQSIQ, General Administration of Quality Supervision, Inspection, and Quarantine of the People's Republic of China; SAC, Standardization Administration of the People's Republic of China; SATCM, State Administration of Traditional Chinese Medicine of the People's Republic of China; CACM, China Association of Chinese Medicine.
